# Technology of Polymer Microtips’ Manufacturing on the Ends of Multi-Mode Optical Fibers

**DOI:** 10.3390/ma13020416

**Published:** 2020-01-16

**Authors:** Monika Żuchowska (Chruściel), Paweł Marć, Iwona Jakubowska, Leszek R. Jaroszewicz

**Affiliations:** Institute of Applied Physics, Faculty of Advanced Technologies and Chemistry, Military University of Technology, 2 gen. S. Kaliskiego St., 00-908 Warsaw, Poland; pawel.marc@wat.edu.pl (P.M.); iwona.jakubowska@wat.edu.pl (I.J.); leszek.jaroszewicz@wat.edu.pl (L.R.J.)

**Keywords:** optical fiber microtip, photopolymerization, material properties, fiber optic element technology

## Abstract

The technology of polymer microtips’ manufacturing on the ends of selected multi-mode fibers has been reported. The study’s key element was an extended description of technology parameters’ influence on the shape of these 3D microstructures. Basic technology parameters such as spectral characteristics of the light source, monomer mixture type, optical power, and exposure time were taken under consideration. Depending on those parameters, different shapes, sizes, and surface structures of microtips were obtained. The spectral characteristics of the light and optical power delivered to a monomer drop were identified as the most important parameters for the formation of the desired 3D shape of the microtip. Presented experimental results are the base for further studies directed to the application of these micro-elements in the fields of optical measurements and sensors’ technology.

## 1. Introduction

The photopolymerization phenomenon has been used in various fields of science and technology because of its unique advantages such as low cost, fast chain reaction, time-efficient, ambient temperature processing, and the possibility of making desirable micrometer-sized 3D structures. Showing only a few examples of industrial implementation, it has found applications in surface fabrication, particle preparation, and continuous flow technology [1]. These materials are present as adhesives, coatings, photoresponsive gels, and photoresists dedicated to microlithography and nanolithography in microelectronics, optoelectronics, holographic data storage, etc. [2]. Application of the photopolymerization phenomenon in the optical fiber technology as a new production method of micrometer sizes’ polymeric structures at the ends of selected optical fibers has been reported. Previously, these polymeric structures, named microtips, were fabricated at single-mode fibers (SMFs) [3,4], including standard telecommunication fiber (SMF-28e+) [5], SMF at 488 nm (SM-450) [6], as well as large mode area photonic crystal fibers (LMA-10) [7]. Their possible applications in microscopy required an investigation of the beam outgoing from the SMF and its distribution in the far-field region [3,4,5], as well as an analysis of the refractive index distribution in this type of optical elements [8].

However, a microtip can be considered as an optical fiber refractive index sensor transducer, as well. Known from the literature, the designs have used optical fiber transducers based on Bragg gratings [9,10], long-period gratings [10,11], plasmonic effects in standard and microstructured optical fibers [10], or even micro-interferometers [10,12]. In the authors’ studies regarding microtips on multi-mode fibers (MMFs), including polymer MMF (GIPOF-62) [6] and silica-based MMF (GIF625) [13], the preliminary results have shown linear changes of return losses when the refractive index (RI) around the microtip was changed within the range of 1.3–1.5. The measured dynamic range of these losses was at the level of 28 dB [13,14]. In this way, it was shown that a microtip manufactured at the end of MMF has a higher back reflecting signal than microtip on SMF. Moreover, microtips produced on optical fibers with a large core diameter have a larger adhesion surface between the microtip base and fiber end face, which reduces the probability of delamination. The two above advantages are the main reason for the investigation of such structures.

In this paper, the extended study presents a technology of polymer microtips’ manufacturing on MMF with a detailed description of the experimental conditions, as well as an extended analysis of the influence of the technology parameters on microtips’ shaping. The main technology parameters selected during the investigation were: composition of monomer mixtures, spectral characteristics of the used light sources, MMFs’ types, deposited mixture quantity, position of the fiber, and energy absorbed by the monomer material during photopolymerization. The last one has been investigated as the composition of two technical parameters, i.e., optical power (P) and exposure time (t). Geometries of manufactured elements were analyzed and compared with different variations of the above-mentioned parameters. Finally, conclusions with a qualitative research commentary and a proposal for potential applications in the optical fiber sensors’ technology were presented. 

## 2. Technology of Microtips on Multi-Mode Optical Fibers

Microtips’ manufacturing procedure consists of two steps. The first step is the application of a liquid monomer mixture as a drop at the end of a cleaved optical fiber [6,13,14] or immersion of the optical fiber into a cuvette with this mixture. In the second step, light propagated in the optical fiber core cures the photopolymer so it becomes a hardened 3D polymer microstructure. The main elements of the technology are: monomer mixture photopolymerization ability, light source radiation parameters, and type of applied optical fiber. Proper selection of these parameters significantly influences the microtip shape. Therefore, the next section of the paper is dedicated to the detailed description of the impact of these parameters on the geometry of these types of micro-optic elements.

### 2.1. Monomer Mixtures and Optical Properties of Polymers

From the huge number of monomers that could be the basis for microtips’ production, the ones that are photopolymerizable were selected. Many tests were carried out, after which two types of multifunctional acrylate monomers met all requirements. In each of the tested mixtures, 3-functional pentaerythritol triacrylate (PETA; Sigma-Aldrich, St. Louis, MO, USA) or 2-functional tricyclo decanedimethanol diacrylate (TCDMA; Sigma-Aldrich, St. Louis, Missouri, United States) were used, and various additives depending on the light source were used in the experiment. Two types of photo-initiating systems (PISs) were used; the mixture was cured with UV (ultraviolet) or VIS (visible) radiation. The UV-curable mixture contained only two compounds, i.e., monomer and photo-initiator. The ranges of possible percentage compositions of mixtures are presented in Table 1.

In this mixture, the PIS 2,2-Dimethoxy-2-phenylacetophenone (DMPAP) was used, which belonged to the α-Dialkoxy-acetophenones’ photo-initiator class [14]. The VIS-curable mixture needs three compounds, i.e., monomer, sensitizer dye, and co-initiator. In this mixture, Eosin Y disodium salt and methyldiethanolamine (MDEA) were used as sensitizer and co-initiator, respectively. Eosin Y is photosensitive in the spectral range from 450 nm to 550 nm and allows use of the trigger photopolymerization process for the used VIS light source [15]. Both above-mentioned chemical compounds were purchased from Sigma-Aldrich. Applications of different spectral ranges of the light sources and various compositions of the monomer mixtures allow obtaining polymers with different refractive indices (RI) [1,16]. In Table 2 are presented measurements of RIs of the above-mentioned polymers. All prepared materials were measured on the Abbe refractometer, where uncertainty is defined at the level of 2σ where σ is the standard deviation of RI.

In all cases, the RI of the prepared mixtures increases after polymerization. Referring them to silica glass for which RIs are of around 1.4607 at 530 nm and around 1.4745 at 365 nm, both polymers have higher RIs. Moreover, the microtip can have an anisotropic structure with a higher RI in the center and with a lower RI in the outer section. However, as was shown by tomographic examinations [8,17], this difference reaches a value of around 0.0007 and the assumption that the RI distribution is homogeneous is acceptable.

### 2.2. Light Sources’ Parameters

In previous papers, the results obtained by using separately coherent light sources [3,13] or UV LED [14] have been presented. In this paper, the influence on the microtip geometry of such source parameters as a full width at half maximum (FWHM) and a central wavelength are investigated. As the light source, the coherent laser at a wavelength of 532 nm and two broadband UV and VIS LEDs were used and compared.

**Figure 1:**
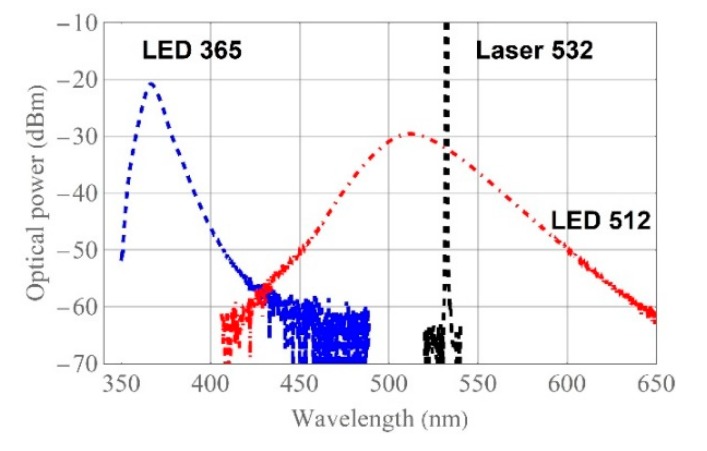
Spectral characteristics of light sources: UV LED, 365 nm (blue curve); VIS LED, 512 nm (red curve); and VIS laser 532 nm (black curve).

Spectral characteristics of the light sources used in all experiments are presented in Figure 1. VIS light sources have central wavelengths at 512 nm, 532 nm for a broadband LED (M530F2, ThorLabs, Newton, New Jersey), and an Nd YAG solid-state laser (Samba, Cobolt, Hubner Group company, Solna, Sweden), respectively. Their calculated FWHMs are of about 20 nm and less than 0.1 nm, respectively. The spectral characteristics of UV LED (M365FP1, ThorLabs, Newton, New Jersey) has a central wavelength of around 365 nm with a 5.0 nm FWHM.

**Figure 2:**
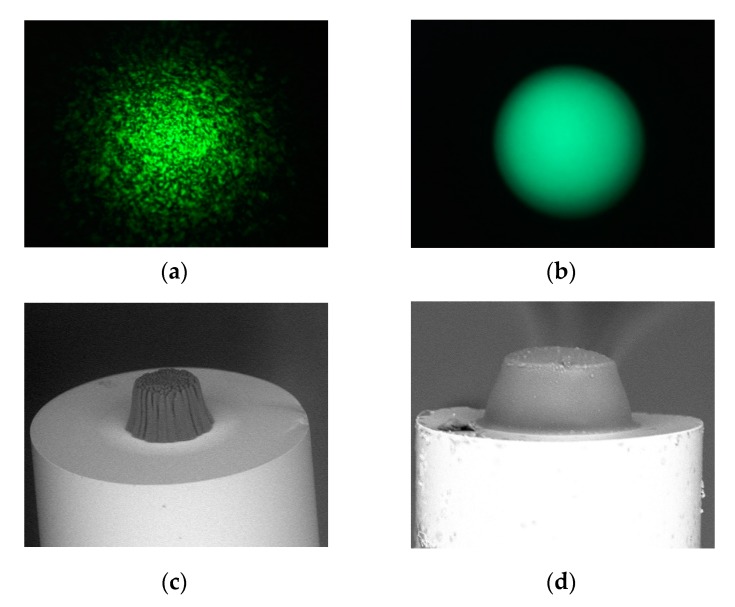
CCD images of the intensity patterns of multi-mode fibers (MMF) with a 62.5 µm core illuminated by VIS: (**a**) laser; (**b**) LED, SEM images of microtips manufactured with mentioned pentaerythritol triacrylate (PETA)-based mixture and prepared with a respective light source, optical power, and exposure time; (**c**) laser, Eosin Y/amine photo-initiating system (PIS), P = 20 µW, t = 30 s; (**d**) LED, Eosin Y/amine PIS, P = 20 µW, t = 60 s.

In Figure 2a,b, examples of intensity patterns in the near field of the fiber output for the standard gradient-index MMF with a 62.5 µm core were shown when the fiber was illuminated by the above-mentioned VIS light sources, whereas in Figure 2c,d, a pair of microtips based on PETA monomer manufactured by using these sources is presented. The surface shape of the manufactured microtips reflects the modal characteristics of the used optical fiber illuminated by given sources. The broadband spectral characteristics of UV LED have a uniform Gaussian-like intensity distribution; the microtip has smooth edges and apex, while the intensity speckle pattern of the narrowband laser remodels these patterns in the 3D polymer structure. Both microtips have quasi-trapezoidal cross-sections, wider in the bottoms (base) and narrower at their tops.

**Figure 3:**
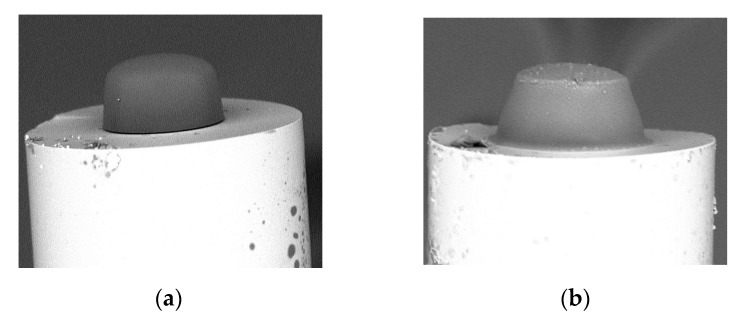
The SEM images of microtips manufactured with mentioned PETA monomer on MMF with a 62.5 µm core illuminated by: (**a**) UV, acetophenone PIS, P = 50 µW, t = 60 s; (**b**) VIS, Eosin Y/amine PIS, P = 20 µW, t = 60 s with respective optical powers and exposure times.

Although the broadband source is key for obtaining a smooth microtip surface [14], the other source parameter has an influence on the general microtip shape. In Figure 3 are shown SEM images of the microtips manufactured by using UV (Figure 3a) and VIS (Figure 3b) LEDs based on a PETA monomer obtained on the same type of MMF as in the previous test. Depending on the used UV or VIS LEDs’ source, the microtip cross-section shape is quasi-rectangular or quasi-trapezoidal, respectively. Additionally, the microtip manufactured by UV light has a rounded apex while the other has a flat one. The above aspect will be deeper discussed in Section 2.4 and summarized in Section 3.

### 2.3. Selected Multi-Mode Optical Fibers

As was shown in the previous section, the optical fiber influences the microtip’s shape due to its modal characteristics. Therefore, different types of optical fibers were selected to optimize the procedure of microtip manufacturing. In this paper were used: gradient-index MMF with a 62.5 µm core diameter and three step-index MMFs with 50 µm, 105 µm, and 200 µm cores diameters. Based on the previous studies, these MMFs were selected in terms of their reflective properties [13,14]. In Table 3, the main parameters of the used MMFs are presented. They were divided into categories related to the core and cladding diameters, numerical aperture (NA), and RI profile distribution. All selected optical fibers were purchased from ThorLabs, and their specific names were presented.

**Figure 4:**
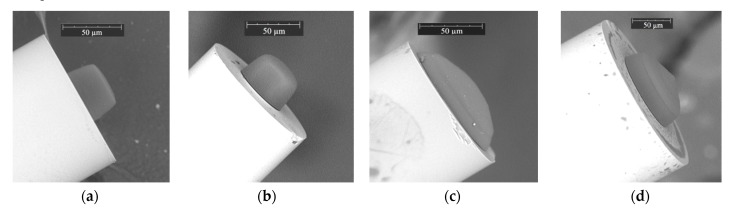
The SEM images of microtips obtained using UV LED with acetophenone PIS on MMF with a respective RI profile, core diameter, optical power, exposure time, and monomer type: (**a**) step-index, 50 µm, P = 5 µW, t = 30 s, PETA; (**b**) gradient-index, 62.5 µm, P = 20 µW, t = 10 s, PETA; (**c**) step-index, 105 µm, P = 30 µW, t = 30 s, PETA; (**d**) step-index, 200 µm, P = 5 µW, t = 60 s, tricyclo decanedimethanol diacrylate (TCDMA).

In Figure 4 are presented the selected microtips obtained using the UV LED and both PETA and TCDMA monomer mixtures at the ends of the MMFs from Table 3. Each of the micro-element was prepared at various optical power and exposure times to show the possibility of shaping this type of optical microstructure.

As demonstrated, all of them have smooth surfaces, but their shapes significantly differ. The microtip on a 50 µm step-index MMF from Figure 4a has a quasi-rectangular cross-section with a flat apex. The microtip on a 62.5 µm gradient-index MMF (Figure 4b) is quasi-rectangular with a rounded apex similar to the microtip in Figure 3a. Increasing the core size of the step-index optical fiber to 105 µm resulted in the shape change to quasi-rounded with high curvature (Figure 4c). Further increase of the core size of the same MMF refractive index profile formed a microtip with a quasi-rectangular cross-section and conical apex (Figure 4d). The above results showed the possibility for microtip shaping by choosing suitable optical fibers.

### 2.4. Technical Parameters of the Manufacturing Process

Technical parameters in the manufacturing process are: position of the fiber, amount of optical energy absorbed by the mixture, and amount of mixture that can be assessed as the droplet size. As an application method of the monomer mixture was used a drop deposition at the end of a cleaved optical fiber by the method previously described [4]. Immersing the optical fiber into a cuvette with the monomer mixture does not give positive results.

**Figure 5:**
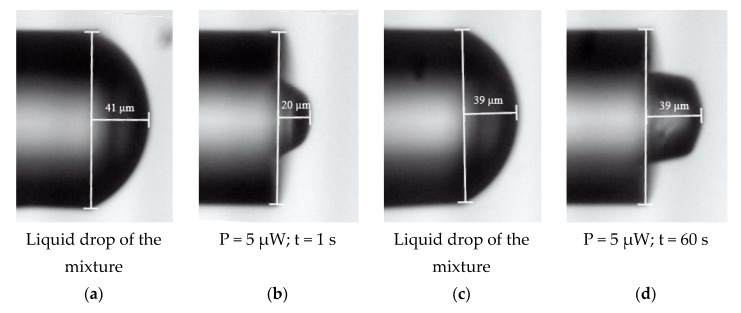
Optical microscope images of the gradient-index MMF with applied PETA monomer mixture drops: (**a**,**c**) as well as microtips formed by VIS LED with Eosin Y/amine PIS for different exposure times and the same source optical power (**b**,**d**).

The shape of the liquid drop deposited at the MMF’s end depends on surface tension forces defined by optical fiber diameter, amount, and viscosity of the monomer mixture [1]. In Figure 5 are shown two pairs of optical microscope images of the optical fiber with deposited monomer drops (Figure 5a,c) and formed microtips for the same optical power and different exposure times (Figure 5b,d). The applied drops of the mixture (Figure 5a,c) have the same height and the same shape because the surface tension forces form a rounded shape on the forehead of the fiber. The photopolymerization process creates a 3D polymer microstructure in the form of a microtip and the height of the microtip varies depending on the absorption by mixture energy, which is proportional to the exposure time, in this example. In the first case (Figure 5b), the short exposure time produces a 20 µm high trapezoidal microtip and in the second case (Figure 5d) the microtip is rectangular at 39 µm high and is equal to the size of the initial drop.

The exposure time, for a given optical power of the source, should be enough to polymerize over the entire area of the applied drop. It results from the fact that the chain process of photopolymerization occurs when the mixture is illuminated with a VIS source, defined in Section 2.1. After radiation stops, the polymerization process is stopped as well. As noted in [18], the cured part of the mixture becomes an extension of the optical fiber core and acts as a waveguide, illuminating and curing subsequent layers of the liquid polymer. Time of exposure does not affect the shape of the edge or the top of the microtip, but it determines its height. Keeping the same optical power, the resulting microtips have the same height as the height of the liquid drop if the exposure time is long enough for polymerization along the entire length of the drop. If the exposure time is too short, not all of the liquid mixture is polymerized, and the microtip has a lower height than the initial drop. For a different testing time of exposure (1 s, 10 s, 20 s, 30 s, 60 s, 120 s), it was found that 60 s is enough to cure the entire height of the drop. Reduced exposure time means less energy delivered to the system, so the manufactured microtip is lower than microtip for longer times.

**Figure 6:**
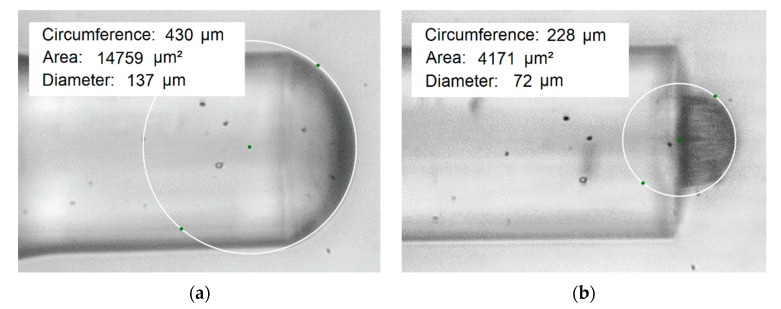
Optical microscope images: (**a**) a drop of liquid polymer; (**b**) microtip cured by using a VIS laser with P = 5 µW and t = 20 s.

Microtips produced on the gradient-index MMF had a slightly rounded apex with a large radius of curvature. The curvature is in proportional relation to the curvature of the liquid drop before photopolymerization and the microtip’s apex radius is always smaller than the drop’s radius (see Figure 6). This discrepancy results from the fact that the polymerization process runs only in a certain area of the drop and the apex shapes are determined by the mode distribution of the light beam on the optical fiber output. This shrinking process of the material occurs when it changes its state from liquid to solid. The experimental results showed that for optical powers within the range of 5 µW–40 µW and exposure times from 1 s to 60 s, the average difference between the curvature radius of the microtip’s apex and drop is of about 26 µm for the VIS light sources and of 36 µm for UV LED. It was previously noted [19] that elements produced on an SMF’s end face had a greater curvature radius than the fiber core diameter, and the microtip curvature radius increased with the exposure time. While for the used MMF, the microtip’s apex curvature radius can be greater or smaller than 62.5 µm and it depends on optical power. Besides, no relationship was found between exposure time and the microtips’ curvature radius. In Figure 6, the microscope images of the liquid drop and microtip with the approximation circles are presented.

The difference between the drop’s curvature and microtip’s apex in the above figures is around 32.5 µm. By careful control of the mixture amount, the microtip height is similar every time. In Table 4, the average microtips’ heights with their uncertainties for an MMF gradient-index with a 62.5 µm core diameter and both monomers’ mixture (PETA, TCDMA) are presented. Moreover, research results have shown that the highest microtip fabricated on an MMF with the core diameter of: 50 µm, 62.5 µm, 105 µm, and 200 µm have lengths of about: 31 µm, 39 µm, 29 µm, and 63 µm, respectively. The position of the optical fiber was always the same, i.e., MMF’s end was directed vertically downward. However, it is worth noting that no differences were found in the microtip’s creation in various fiber settings in space. What is most important here is that the adhesion force between the fiber and the polymer drops while the gravity force is of secondary importance.

**Figure 7:**
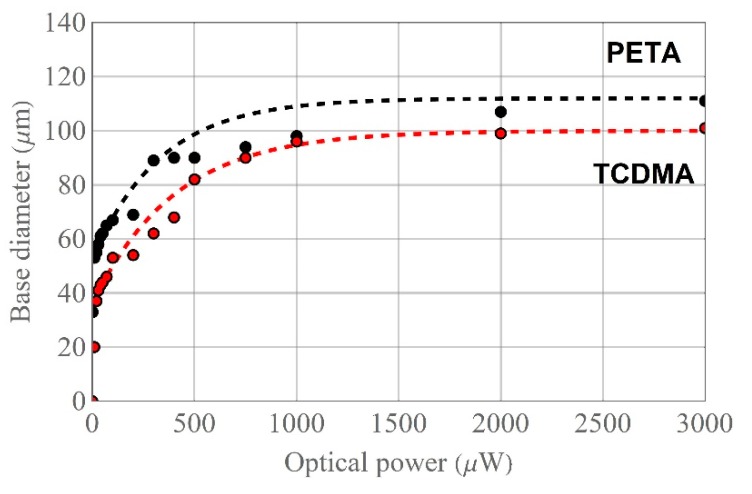
Microtip base diameter vs. optical power of VIS laser with Eosin Y/amine PIS on the gradient-index MMF with the mixture based on monomers: PETA (black dots) and TCDMA (red dots) for t = 60 s with respective approximation curves (dashed lines).

During the experiment, it was found that absorbed optical energy is a more important parameter. This energy is defined by optical power and exposure time. Depending on this parameter, microtips have different base sizes [13,14]. Moreover, it has been noticed that with the optical power increase, the microtip base diameter increases as well. The rate of this change depends on the monomer mixture composition and the source spectral characteristics. In Figure 7 the results for microtips, based on mixtures with PETA (black dots) and TCDMA (red dots) monomers, manufactured by VIS laser are presented.

SEM images of microtips allow measuring the size of their bases. Analysis of the data related to the PETA-based microtips (black) indicates that, theoretically, the microtip base covers the entire core of the MMF at the optical power of about 30 μW (black curve approximation) while the experimental value is of around 40 μW. For the mixture with the TCDMA monomer, the approximated optical power value of 150 μW is enough to form a microtip with a base diameter similar to the core diameter of the used MMF (red curve), while the experiment’s obtained value (red dots) was of 300 μW.

## 3. Summary of Microtips’ Geometry Shaping on the MMF

**Figure 8:**
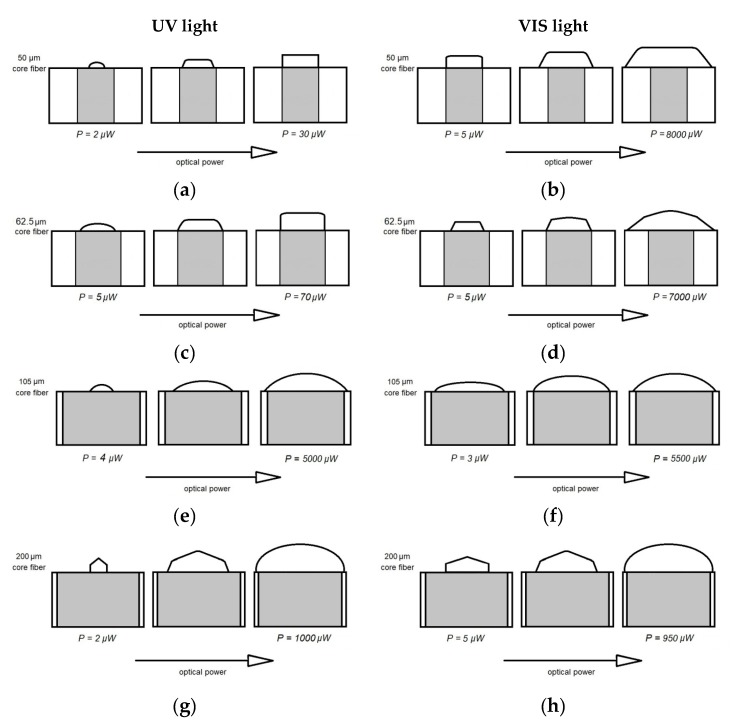
Shapes of microtips’ evolution obtained by using UV light with acetophenone PIS and VIS light with Eosin Y/amine PIS on various optical fibers with a core diameter: (**a**,**b**) 50 µm; (**c**,**d**) 62.5 µm; (**e**,**f**) 105 µm; (**g**,**h**) 200 µm.

In the previous section, the main parameters of the microtips’ manufacturing technology were identified and described. The graphical summary of the microtip shaping possibilities on the selected MMFs is shown in Figure 8. The evolution of the microtips’ cross-sections’ shape is presented in the form of sketches. Each sketch is related to the selected optical power ranges for all tested MMFs, separately. Evidently, the shape is connected with the used light source type, as well as the technical parameters of the MMF.

The microtip produced by VIS light (right column, Figure 8b,d) had a cross-section that can be approximated as trapezoidal, and the microtip produced by UV light (left column, Figure 8a,c) had a cross-section of the rectangular shape. The reasons can be in the light transition and different refraction angles between the MMF and the applied drop of the monomer mixture. It is a transition from a material with a lower refractive index to a material with a higher refractive index (Table 2). As a result, various of the cross-sections’ shapes were obtained.

The microtip formed on a step-index MMF with a 50 µm core diameter by UV light has a hemispherical shape, and its cross-section is semi-circular with a small optical power (P = 2 µW). With the increase of the optical power, it tends to a rectangular shape (Figure 8a). The microtip at the same MMF but manufactured with VIS light has a quasi-rectangular cross-section and evolves with an increase of the optical power to a trapezoidal shape (Figure 8b). The micro-element formed on a gradient-index MMF with a 62.5 µm core diameter (Figure 8c) has a semi-circular cross-section with a large radius of the curvature comparable to a plano-convex lens. Higher values of optical power change its cross-section to rectangular. The microtip formed on this type of MMF using VIS light changes from trapezoidal to a semi-circular cross-section with the increase of the optical power (Figure 8d). The microtip formed on a step-index MMF with a 105 µm core diameter has a similar semi-circular cross-section for both light sources, and with increasing optical power, the manufactured microtips increase (Figure 8e,f). Finally, the microtip on a step-index MMF with a 200 µm core diameter created by using both types of light sources has an axiconal and semi-circular cross-section. The conical top for the starting optical power becomes hemispherical with the power increase (Figure 8g,h). This fact should be noticed so all the above-described optical elements have similar geometries regardless of the type of monomer used for the mixture.

**Figure 9:**
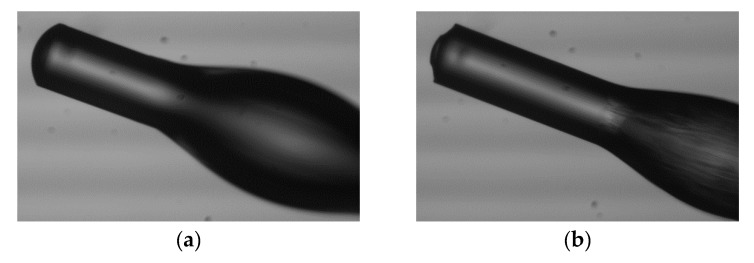
Optical microscope images of the optical fiber with deposited monomer mixture (**a**); polymer flange produced by the back-reflected light on gradient-index MMF by VIS laser with Eosin Y/amine PIS (**b**).

One of the most spectacular side effects of the photopolymerization process on the MMF is a polymer flange formed on the outer side of the optical fiber (Figure 9). It is created by the polymerization of the monomer mixture deposited on the side of the MMF by back-reflected light at the interface of cleaved optical fiber and microtip.

The produced polymer flange on the MMF reflects the intensity pattern of the modal characteristics of the used optical fibers. The polymer flange has a grooved surface and oval cross-sections.

## 4. Conclusions

From a technological point of view, the geometry of polymer microtips at the end of the MMF depends on a monomer mixture composition, light sources’ parameters including the amount of delivered energy and spectral characteristics, type of used optical fiber, as well as the size of the liquid drop, and optical fiber position.

Microtips manufactured on chosen the MMFs had a large reflective surface and better optic properties compared to those manufactured on SMFs. However, the fiber modal structure connected with the used light source has an influence on the manufactured microtips, whose effect is not observed for SMFs. Therefore, a light source with a broad spectrum is preferred. When the beam has a wider spectrum, more modes are propagated inside the fiber, and thus the microtip surface becomes smooth. The used mixtures varied depending on the added monomer and the initiator type. By changing the mixture’s composition changes, in its RI could be observed.

Two main technical parameters of the manufacturing process are optical power and exposure time. They determine the amount of delivered optical energy to the monomer drop. It was noticed that the microtip base diameter increases with the increase of the optical power, while the exposure time influences the microtip height. The adhesion forces between the optical fiber end and the polymer drop are greater than gravity forces, so the optical fiber position does not significantly affect the creation of the microtip.

Based on the presented analysis, an optimal manufacturing process of the microtips was obtained. These efforts have been made to gather knowledge on how to plan the strategy of such kinds of 3D microstructure formation in order to reach the required optical properties. All the above-presented experimental results are considered in their applications’ point of view. Measurements of reflection and transmission properties of manufactured polymer microtips allow us to validate and indicate how to select process parameters to obtain the proper geometry of this element. Reflection measurements were previously performed and have led to testing such optical elements as optical fiber RI sensors’ transducers. Transmission properties have been pre-characterized, and for the tested microtips, their output intensity patterns are comparable to divergent light. The microtip increases numerical aperture and can be considered as an illuminating point source dedicated to optical measurements. However, these properties will be studied in the future.

## Figures and Tables

**Table 1:** The measured value of the tested mixtures’ refractive index (RI).

Mixture Components	UV Mixture	VIS Mixture
Monomer (wt %)	99.70–99.95	89.00–92.40
Sensitizer (wt %)	0.05–0.30	0.40–1.00
Co-initiator (wt %)	-	7.00–10.00

**Table 2:** The measured value of the tested mixtures’ refractive index (RI).

-	UV Mixture				VIS Mixture			
Material	PETA liquid	PETA solid	TCDMA liquid	TCDMA solid	PETA liquid	PETA solid	TCDMA liquid	TCDMA solid
RI	1.4834	1.5159	1.5041	1.5077	1.4816	1.5076	1.5012	1.5268
Uncertainty of RI	0.0004	0.0002	0.0004	0.0008	0.0006	0.0008	0.0004	0.0004

**Table 3:** Main technical parameters of selected MMFs used in the experiments.

MMF Catalog Name	Core Diameter	Cladding Diameter	NA	RI Profile
FG050UGA	50 µm	125 µm	0.22	step-index
GIF625	62.5 µm	125 µm	0.275	gradient-index
FG105LCA	105 µm	125 µm	0.22	step-index
FT200EMT	200 µm	225 µm	0.39	step-index

**Table 4:** Average heights of microtips with their uncertainties for optical power range from P = 5 µW to P = 40 µW and time of exposure from t = 1 s to t = 60 s.

-	VIS Laser		VIS LED		UV LED	
**Monomer**	PETA	TCDMA	PETA	TCDMA	PETA	TCDMA
**Average Height (µm)**	33 ± 3	25 ± 2	29 ± 4	-	24 ± 4	27 ± 5
